# “Ngany Kamam, I Speak Truly”: First-Person Accounts of Aboriginal Youth Voices in Mental Health Service Reform

**DOI:** 10.3390/ijerph20116019

**Published:** 2023-05-31

**Authors:** Hunter Culbong, Ashton Ramirez-Watkins, Shae Anderson, Tiana Culbong, Nikayla Crisp, Glenn Pearson, Ashleigh Lin, Michael Wright

**Affiliations:** 1 College of Arts and Social Sciences, Australian National University, Canberra 2601, Australia; hunter.culbong@anu.edu.au; 2 Youth Programs, Port Hedland Local Council, Port Hedland 6721, Australia; ashton.ramirezwatkins@porthedland.wa.gov.au; 3 Kulbardi Aboriginal Centre, Murdoch University, Perth 6150, Australia; shae.anderson@murdoch.edu.au; 4 School of Nursing and Midwifery, The University of Technology, Sydney 2000, Australia; tianalea.culbong@student.uts.edu.au; 5 Design Participation and Inclusion, Neami National, Perth 6000, Australia; nikayla.crisp@neaminational.org.au; 6 Telethon Kids Institute, University of Western Australia, Perth 6000, Australia; glenn.pearson@telethonkids.org.au (G.P.); ashleigh.lin@telethonkids.org.au (A.L.); 7 School of Allied Health, Faculty of Health Sciences, Curtin University, Bentley 6102, Australia

**Keywords:** co-design, consumer participation, Indigenous, lived experience, mental health service, youth leadership, wellbeing, Aboriginal youth, First Nations, youth voice

## Abstract

Aboriginal young people are experts in their own experience and are best placed to identify the solutions to their mental health and wellbeing needs. Given that Aboriginal young people experience high rates of mental health concerns and are less likely than non-Indigenous young people to access mental health services, co-design and evaluation of appropriate mental health care is a priority. Increasing Aboriginal young people’s participation in mental health service reform is key to ensuring services are culturally secure, relevant and accessible. This paper presents first-person accounts from three Aboriginal young people who worked alongside their Elders and in a positive and constructive partnership with mainstream mental health services on a three-year participatory action research project in Perth, Western Australia, in Whadjuk Nyoongar *boodja* (Country). The young people recount their experiences as participants and co-researchers on a systems change mental health research project and share their views on the importance of privileging Aboriginal youth voices. Their accounts highlight that Aboriginal young people’s participation and leadership must be understood through a decolonising lens and that working in genuine partnership with the community is key to increasing their contact and engagement with mental health care and improving mental health and wellbeing outcomes.

## 1. Introduction

### 1.1. Decolonising Youth Mental Health Services

Strengthening Australian Aboriginal young people’s voices in youth mental health service provision is key to decolonising mainstream services and improving mental health and wellbeing outcomes. Decolonisation, when understood through an Aboriginal worldview lens, involves the reconnection of culture, community and land to reinstate and privilege Aboriginal voices and viewpoints. Decolonising efforts are pursued by Aboriginal people in ways that reclaim power, Indigeneity and ways of working. [1,2], Aboriginal youth co-researcher and co-author, Ashton Ramirez-Watkins (A.R.-W.) shares his reflections on the intersection between an Aboriginal youth voice and the ongoing impacts of colonisation on Aboriginal young people, families and communities:

*From an early age, I (A.R.-W.) was taught about the historical impacts that affected Aboriginal people in Australian society. This helped shape my worldview on oppression and discrimination from a young age, whilst being taught to be firm in your views and moral beliefs. I see this as the foundation for what became the strength in finding my voice to speak out on the oppression that Aboriginal people face in society. Throughout my adolescence, I was challenged constantly about my beliefs and views on Aboriginal people in relation to Australia. This made me want to find out my people’s truth and empower myself with knowledge about Australian Aboriginal society. This drive to seek the truth revealed to me why Aboriginal people are truly in the predicament that they are in through researching about the black history of Australia. Seeing how everything that made an Aboriginal person was stripped, dispossessed, assimilated and denied throughout society, systematically creating a social structure of disempowerment and suppression. This social structure was made by non-Aboriginal people to benefit non-Aboriginal people. Aboriginal people’s opinions or voices were never included throughout any decision-making processes, and now Aboriginal people are suffering from mental health as a whole community. So it is the voice of Aboriginal and Torres Strait Islander people that need to be heard. When services deal with Aboriginal and Torres Strait Islander young people, their opinion and voice need to be included in service delivery and decision-making processes that impact their lives. This power relation is usually in the form of “youth groups”, but decision-making is never really inclusive of young people within services. Usually, it is team members engaging in community consultation about decisions already made in meetings for young Aboriginal and Torres Strait Islander people.*

Australia’s colonial history and assiduous silencing and disempowering of Aboriginal peoples, including Aboriginal young people, who are often further disempowered due to their age, illuminates the critical importance of decolonising approaches. Increasing Aboriginal young people’s participation in mental health service design and delivery is key to understanding their needs and ensuring services are culturally secure, relevant and accessible [3,4]. The Australian National Standards for Mental Health Services specify that consumers and carers be actively involved in the development, planning, delivery and evaluation of services [5]. There is strong evidence that youth-specific mental health services will be more efficient and effective if young people are meaningfully engaged in the design of mental health support services [6,7,8,9,10,11,12,13]. Despite the past decade of research in this area, there remain inconsistencies between the theory and practice of youth participation in the Australian mental health sector and, in particular, the participation of Aboriginal young people.

Improving the accessibility and responsiveness of youth mental health services is critical, with suicide remaining the leading cause of death for Australian Aboriginal young people aged between 15 and 34, with rates over three times that of non-Aboriginal young people of the same age [14]. Aboriginal young people experience high rates of mental health concerns but are less likely than non-Aboriginal young people to access mental health services, are more likely to present in crisis or at a chronic stage, and often engage with services for shorter periods of time [15,16]. Their limited engagement is typically attributed to Aboriginal young people’s lack of trust in mainstream services, non-Aboriginal service providers’ lack of understanding about Aboriginal people and culture, and inflexible service models that do not meet the needs of Aboriginal young people, families and communities [3,4,17]. Although Aboriginal youth mental health is a priority for the government [18], the disproportionately high suicide rates and overall poor mental health outcomes indicate that current government-led solutions are not effective and greater community-led solutions are required [3,4,19]. It is critical that the mental health sector moves towards a way of working that places Aboriginal young people’s voices at the centre, where their lived experience is recognised as an invaluable resource to developing culturally safe mental health care and improving mental health and wellbeing outcomes, in terms defined by them [3,4,11,20,21,22]. 

This paper explores the impact of Aboriginal young people’s voices on mental health service reform, drawing on learnings from The Building Bridges Project: Co-designing engagement with Aboriginal youth (Building Bridges) [3,4]. The project aimed to develop meaningful partnerships between Aboriginal young people, their Elders and youth mental health service providers in the metropolitan area of Perth, Western Australia. A key outcome of the project was the co-design of a work practice model shaped by the cultural knowledge and ways of working of the Elder and youth co-researchers [3,4]. 

The aim of the paper is to place the voices of Aboriginal and Torres Strait Islander youth centrally, and as such, we do not present findings from service providers or Elders, as these have been documented elsewhere [3,4]. The paper is structured around six themes. As per cultural protocol, we begin by acknowledging the *boodja* (Country) and the local community context in which this research was conducted. Second, we position ourselves as authors as a group of Aboriginal and non-Aboriginal academics and Aboriginal youth co-researchers. Third, we provide background to the project’s participatory action research methodology and co-design process. Fourth, we discuss the first-person account approach and the methods employed in co-writing this paper. Fifth, we present first-person accounts from three youth co-researchers about the experience of sharing their voices within the Building Bridges project. Finally, we discuss the implications of engaging Aboriginal youth voices for mental health sector reform to close the gap on Aboriginal youth mental health and wellbeing outcomes. 

### 1.2. Local Context: Whadjuk Nyoongar Boodja 

**Figure 1:**
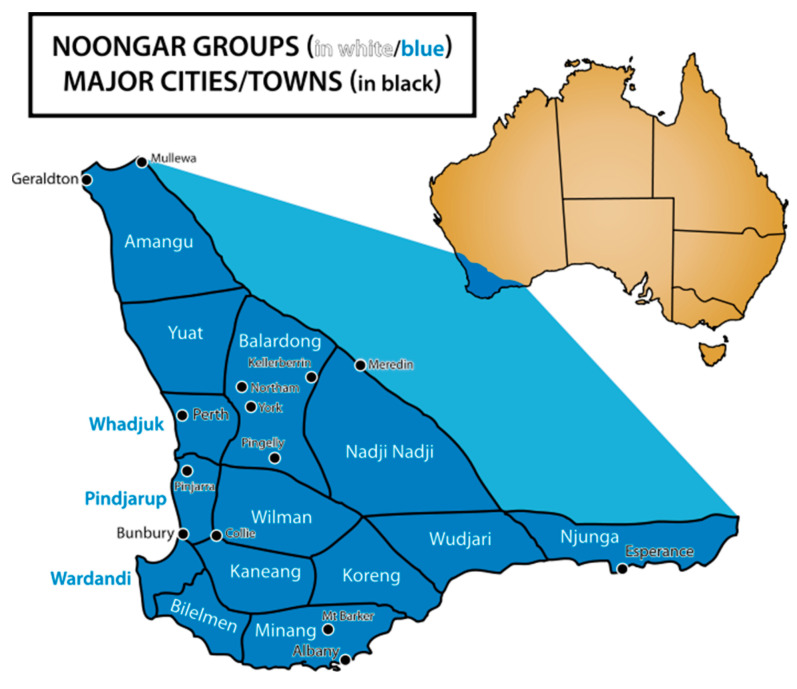
Map of Nyoongar boodja in the southwest region of Western Australia (image by Brooke Ottley, 2011. Source: Wikimedia Commons, available online: https://commons.wikimedia.org/wiki/File:Noongar_regions_map.svg (accessed on 17 April 2023).

Australia’s First Nations peoples are culturally diverse, with Aboriginal and Torres Strait Islander people choosing to be recognised for their uniqueness and diversity rather than as a homogenous group. Whilst this research may have implications for Australian First Nations communities and globally, it is important to ground this work in its local context and place. The Building Bridges project was located in *Whadjuk Nyoongar boodja*. *Nyoongar boodja* covers the south-western portion of Western Australia and Nyoongar people are one of the largest Aboriginal cultural groups in Australia [23]. *Whadjuk* is one of fourteen clan or language groups that make up the Nyoongar Nation (Figure 1) and Whadjuk people are the traditional custodians of the land where the capital city of Perth (known to Nyoongar as *Boorloo*) is located. Nyoongar culture and history are rich, and Nyoongar people have maintained a continuous connection to their boodja for over 45,000 years [23]. As is the case in many Indigenous cultures, Nyoongar Elders are teachers, storytellers, language holders, healers and historians in their own right. They carry the knowledge, practice and spirit of their culture forward into the next generation [24,25,26,27]. As per cultural protocol, Nyoongar Elders provided leadership to the Building Bridges project and grounded the research in Nyoongar culture and *kaartdijin* (knowledge). 

### 1.3. Situating the Authors

As well as foregrounding the land on which this research was conducted, it is necessary to position the authors. This aligns with cultural ways of connecting; introducing who we are and where we come from [1,28,29]. The authors of this paper include the Aboriginal and non-Aboriginal researchers and three Aboriginal young people who were engaged as participants and co-researchers, recognising their role as partners in the research and their contributions to both the project outcomes and its shared, collaborative, co-design processes. While academic journal articles are often written in the third person, we will use the first person “I (initials)” when one author is speaking as an individual, and “we/our” for the collective group of authors. As this research is Aboriginal-led and aims to privilege Aboriginal voices, the use of “we/our” is positioned from an Aboriginal perspective [30,31]. 

### 1.4. Youth Co-Researchers’ Voices

*I (S.A.) am a Yamatji and Wongi woman living and working in Boorloo on Noongar boodja. I am driven by my passion to improve mental health outcomes for Aboriginal and Torres Strait Islander youth and I strongly believe in the important role that culture and connection plays in healing our people. I am currently the Engagement and Communications Coordinator for Kulbardi Aboriginal Centre, which sits on Murdoch University’s South Street campus. My role enables me to connect with our community whilst empowering our mob to further their education and become leaders in their chosen industry or discipline. I am also currently a student myself, studying a double degree in Community Development and Psychology.*

*I (A.R.-W.) am a Kariyarra, Ngarluma and Whadjuk man from Perth and was a youth co-researcher on the project. My understanding of local Indigenous culture is deep because of my background and connection to culture. I was born in Whadjuk Nyoongar Budja (country) at King Edward Hospital Subiaco in 1994. My mother and father were born in Port Hedland and grew up being “Mulbra”s” (Aboriginal). I am currently studying a Bachelor of Youth Work at Edith Cowan University. I have completed mentoring in the Kimberley and Pilbara regions and found the experience to be very rewarding and hope to become a leader in my community.*

*I (H.C.) am a 19-year-old Nyoongar man from the southwest of Western Australia. I was a youth co-researcher on the project and am currently studying a double degree in Criminology and International Security at Australian National University. I believe mental health is very important for youth. Young people are vulnerable and at risk of developing problems that can affect them for the rest of their lives. We need to create safe and healthy communities where young people are able to access the services they need.*

### 1.5. Research Team’s Voices

*I (M.W.) am a Yuat Nyoongar man and my ancestral family are from the Moora and New Norcia area of Western Australia. I previously worked as a hospital social worker and a manager of an Aboriginal mental health service. I have conducted research over several years exploring solutions to the poor relationships between mental health and drug and alcohol services and Aboriginal peoples, and was the Chief Investigator of the Building Bridges project. I provided research and cultural leadership to the project and oversaw the development of this paper.*

*I (G.P.) am a Nyoongar man and my mother was a member of the Stolen Generation.* (The ‘Stolen Generation’ refers to the Aboriginal and Torres Strait Islander children who were forcibly removed from their families by Australian Federal and State government agencies and church missions through a policy of assimilation. The then Human Rights and Equal Opportunity Commission (now the Australian Human Rights Commission) conducted a National Inquiry into the separation of Aboriginal and Torres Strait Islander children from their families and the landmark report, *Bringing Them Home*, was tabled in federal Parliament on 26 May 1997.)*. I am the Head of Aboriginal Research at the Telethon Kids Institute. I lead the Kulunga (Child) Aboriginal Research Development Unit and am a member of the Institute Leadership Team. As part of my role, I ensure the Institute’s research reflects the needs of Aboriginal families and that research is conducted in accord with Aboriginal community ethical and cultural protocols. I am also a trained primary school teacher. I am currently completing a Doctorate at the University of Western Australia and my research project has explored delivery of child protection, child health and educational services to Aboriginal families. I was an Investigator on Building Bridges and provided overall leadership to the project.*

*I (A.L.) am a wadjella (white) woman born in Johannesburg, South Africa. I moved to Perth, Australia when I was 11. After living over east and overseas for many years, I returned to Perth 6 years ago. I am passionate about youth mental health and equity in the provision of mental health care for all young people. I was an Investigator on Building Bridges and provided leadership and mentorship throughout the project and provided input into the development of this paper.*

*I (T.C.) am a Nyoongar woman from the southwest of Western Australia. I have worked with Aboriginal young people for over 10 years and have completed a Master of Public Health at Deakin University. I am passionate about my community and ensuring our young people are provided the opportunity to participate in decision-making processes. In my role as Research Associate I brought experience in Aboriginal community and youth engagement to Building Bridges, and worked very closely with the youth co-researchers throughout the project and in the development of this paper.*

*I (N.C.) am a wadjella (white) woman and was born and raised in Perth, Western Australia. My mother was born in England and on my father’s side I am the fourth generation born in Western Australia. My family origins are in England and Scotland. I have a background in Psychology and previously worked as a youth mental health clinician. I am passionate about mental health and youth leadership, and it has been a privilege to build lasting relationships with Aboriginal Elders, young people and community members through the Building Bridges project. As a Research Associate, I supported project coordination and also worked closely with the youth co-researchers to develop this paper.*

## 2. Materials and Methods

### 2.1. Participants/Co-Researchers 

The 3 Aboriginal young people co-authoring this paper were part of a larger group of 10 youth co-researchers (aged 16 to 25) engaged in the Building Bridges project, alongside seven Nyoongar Elders, recruited through community information sessions and snowball sampling through community connections. During the 3 year project, this group of Elder and youth co-researchers worked directly with staff from 3 mainstream youth mental health services, including 17 non-Aboriginal staff and seven Aboriginal staff, located in Perth, Western Australia. Their roles included senior managers, team leaders, mental health clinicians, community engagement workers, and administrative staff. Policy and advocacy representatives from key organisations across the mental health, youth health and Aboriginal health sectors were also engaged to support the project translation, including eight non-Aboriginal policy officers and one Aboriginal policy officer [4].

### 2.2. Aboriginal Participatory Action Research 

The Building Bridges project applied an Aboriginal participatory action research approach [1,2,3,31,32,33,34,35,36,37]. Aboriginal participatory action research involves community collaborative practices and local knowledge systems that are “dynamic, constantly evolving, influenced by a community’s pragmatic, creative and experimental responses to the internal and external social, political, cultural and environmental stressors they experience” [2]. This methodology allowed for the development of shared spaces where different knowledge systems could be valued and exchanged. The research process was an intervention in itself and aimed to engage, foreground and empower “silenced” voices [38] and resists the disempowering impacts of colonisation and systemic racism [2]. Similarly, youth participatory research approaches are systematic subversive exertions on the dominant structures that silence and disempower young people, in particular Aboriginal young people who directly feel the impacts of colonisation. The challenge is to sustain decolonising efforts that risk the tokenistic participation of Aboriginal youth.

Research and engagement activities over the three years were iterative, with each phase building upon previous phases. The project initially applied two Aboriginal research principles, namely: going On Country and engaging in Shared Storying [1,4]. These activities aimed to prepare and engage non-Aboriginal participants in cultural ways of relating and sharing knowledge to deepen their understanding of a Nyoongar worldview [3,4,17,39]. The research team then facilitated an in-depth co-design process to identify what work practice changes were required to better meet the needs of Aboriginal young people and improve their engagement with mainstream mental health services. The co-design phase included three co-design workshops involving the research team, Elders, young people, service staff and policy staff. Service-specific working groups were then established to work on more targeted goals. This included groups of two Elders and two to three young people working closely with the members of each specific service for up to twelve months. As the young people were paired with different services for this phase, their experience was unique to that service partnership. While participatory principles were employed throughout, it is the process of engaging youth voices that is the focus of this paper [40].

### 2.3. First-Person Accounts Approach

This paper presents our shared learnings, as researchers and co-researchers, and as academics and community members, about the inclusion of Aboriginal young people’s voices in the Building Bridges project, as a key driver in mental health service reform. To achieve this, we selected a first-person account approach to create space for the youth co-researchers to speak for themselves as experts in their own experience and as a demonstration of the need to trust young people to assert their voices and exert control and ownership over the process and outcome. A first-person account is considered a culturally appropriate methodology as it contextualises research findings for the benefit of Aboriginal young people and preserves community narratives and voices [41,42]. First-person accounts align strongly with an Aboriginal participatory action research methodology and reflect the co-design principles of the project. The collaborative paper development process provided youth co-researchers with a deeper understanding of academia and publication writing and the potential for their local experiences to have a global impact. It also grounded the research team in their process of weaving together academic and experiential knowledge, actively making space for Aboriginal young people’s stories and experiences. Similar to the project overall, the co-writing process was relationship-based. The feedback loop between the research team and youth co-researchers built mutual trust, respect, reciprocity and relevance into the process [3,4,38].

#### Research Question

For the purposes of developing a publication, the broad topic of “youth voice” was collaboratively identified and each youth co-researcher provided a written response to the following question: “Why are Aboriginal young people’s voices important? Youth co-researchers were also asked: Can you share your experience of the Building Bridges project, in particular, your experience of expressing your voice as a young person in the space?” Participants had the opportunity to edit their accounts throughout the process to ensure they felt comfortable with how they were being represented. Facilitated by the research team, the group of young people then met to review each other’s accounts, explore similarities and differences between each of their pieces, and identify key themes. This dialogue aimed to ensure the narrative of the paper, as a whole, was grounded in the young people’s knowledge, ideas and interpretations. The research team initially drafted the introduction, method and discussion, and the full manuscript was edited by all the youth co-researchers. Each young person provided informed consent for their accounts to be included in this paper with their names and other identifying information included (ethics approval by the Western Australian Aboriginal Health Ethics Committee, HRE762). The youth co-researchers were financially remunerated for their participation. The following section presents the first-person accounts of each of the youth co-researchers, followed by a discussion about the implications of their insights and experiences.

## 3. First-Person Accounts

### 3.1. Straight Talk: Shae Anderson (S.A.), Youth Co-Researcher

In 2018, I (S.A.) completed a national Indigenous women’s leadership program called Straight Talk. The program challenged participants to consciously step into leadership roles where the presence of Aboriginal and Torres Strait Islander people, particularly women, is rarely felt. This empowered mindset is the one I challenged myself to enter Building Bridges with – to speak up in a space where I typically would not feel comfortable to even voice my opinion, let alone to challenge the delivery of mental health services. 

Having accessed youth mental health services myself in the past, I knew there were gaps in the system which needed addressing, so to be invited into a space that has the potential to drive real change is momentous. We cannot underestimate the value and richness that Indigenous perspectives, experiences, and ways of working bring to the table, and I think this rings especially true for this project. 

To the project team: You have all been fierce advocates for the youth co-researchers throughout this experience and have ensured our perspectives were not only heard but valued. Thank you for your dedication, passion and guidance—Building Bridges would not have been the experience it was without you.

To our Elders: You are such beacons of strength, knowledge and passion, and I am so grateful to be able to walk in your footsteps. Thank you for investing in us throughout this journey and for giving your wisdom and guidance so freely. You have instilled in us the confidence to step up as leaders and to continue the crucial work of advocating for our community, particularly in the youth mental health sector.

To my fellow youth co-researchers: I was lucky enough to be connected to most of you before the project in some capacity, however, this experience has given me a newfound respect and admiration of you all. To be in a room full of people being led by young Aboriginal and Torres Strait Islander voices was an experience I will never forget. If there is anything the past few years has taught me, it is that this is not just a project to us—this is our reality. The people accessing these services are our friends, our family and ourselves, hence why projects like Building Bridges are so crucial. I am so incredibly proud to work with such inspirational and passionate young people and I cannot wait to see where this experience takes us next.

To the service providers: Thank you for taking up this challenge. It is one thing to commit to a project like Building Bridges on paper, but another thing entirely to invite and accept criticism, to listen to the unique experiences and challenges of a minority group and to absorb it all with a view to creating institution-wide change.

We have come a long way, but we have so much further to go. I hope you can all take comfort in knowing that you are not walking this journey alone as you have some incredible young leaders walking alongside you.

### 3.2. Ngany Kamam (I speak truly): Ashton Ramirez-Watkins (A.R.-W.), Youth Co-Researcher

I (A.R.-W.) think one of my favourite experiences during the project was the time us young people started to actually realise why it is so important to be a part of this… to have this communication with services and why there is a need for the young’s people voice to be heard during these meetings and why there needs to be a better approach towards mental health, between the young people and also and an Aboriginal community. This opportunity provided a platform for inclusive, reflective practice about cultural competence in service delivery. Throughout this process, I have realised the importance of having a young Aboriginal and Torres Strait Islander voice in mental health service meetings.

One of the many experiences gained through working with Building Bridges was being able to practice cultural ways of working, through having Elders teach younger generations the needs that the Aboriginal community demands. Also, having the opportunity to debrief and reflect as a group to discuss challenges as a group, created initiatives to help empower the voice of the young people during meetings. Some challenges included the need to address the group dynamics of cultural authority between Elders and the young people. It was these challenges that helped create an understanding of the need to be reflective when discussing different ways of working.

This way of working helped bring different trains of thought in meetings and provided a collaborative approach when facing issues that service providers deal with. Building Bridges helped create a process where a young person’s voice is heard from a top-down approach, providing an innovative and empowering model for service providers to work with in future practices. I think the biggest lesson is putting faith and invest in their time with the young people to become leaders within communities to really get the support from Elders and older people to really give the young people a voice where it can be heard and can be given strength, that’s probably the most important thing.

When the colonial laws and systems were made for Aboriginal and Torres Strait Islander people, it has never been the voice of an Aboriginal or Torres Strait Islander person who has made the decisions for their community throughout history. So, when the laws and systems were made for our young people, it still has not been the voice of our young people in the decision-making processes. Only when their voice is heard can we know as a society what is really needed for the empowerment of our next generation of leaders.

### 3.3. A Call to Action: Hunter Culbong (H.C.), Youth Co-Researcher

The Building Bridges project has been an enriching experience that provided me (H.C.) with knowledge and an opportunity to give back to my community. I have long desired to contribute to the Aboriginal community and Building Bridges has allowed me to do so in a progressive manner. Through this experience, I have developed insights into mental health, the mental health sector and cooperative ways of working, which have been highly valuable. It has allowed me to be educated on mental health issues, which affect the Aboriginal community while also refining my ability to give back and assist.

Developing and conveying my voice in this space as a young man proved to be a challenging process but ultimately a rewarding and fulfilling experience. The combination of mental health professionals, policymakers and Aboriginal Elders created an intimidating environment where it sometimes became difficult to express my ideas. The knowledge and wisdom from the other groups within this space come across as more valued. As a young person who does not hold as much life, cultural or professional experience, it was difficult to contribute much in the early stages. Instead, I tried to learn and understand the perspectives of the other groups within the project in order to shape my own ideas about mental health. 

Because the project was shaped around young Aboriginal people, I found that the knowledge and experiences we put forward were important because we were the target demographic the project sought to understand. Because of this, I found expressing myself became less challenging, as I could relate to the experiences of other Aboriginal youth and contextualise that to the Elders, mental health professionals and policymakers. The discussion space looked to me like a synthesis of everyone in the project relaying their own ideas and experiences. I found that my ideas and experiences, along with those of the other young people, fit into that space and was vital to the discussion.

By connecting with Aboriginal people, services can comprehend the serious issues young people face by understanding the cultural differences and build trust and rapport in the Aboriginal community to better help Aboriginal youth access services they need. Collaborating with young people is essential in developing service plans that are orientated towards their needs, while allowing young people to expand their voice and perspective on issues that affect them.

Building Bridges provided Aboriginal people and services with the opportunity to help develop a way of working to better Aboriginal health by truly understanding Aboriginal people and culture and can be an example to other public entities in working for the advancement of Aboriginal people. I believe that if the justice system, health system, education system or any other services can implement a model such as the one Building Bridges has developed, in which engagement with Aboriginal people and communities is valued as a key component of working, and acknowledging the differences Aboriginal people face in our society, then we can bridge the societal gap Aboriginal people have in our path. I call on services to work with Aboriginal people to integrate a way of working, which interacts and values an Aboriginal contribution and perspective.

## 4. Discussion: Implications of Sharing Aboriginal Young People’s Insights

These three first-person accounts provide invaluable and situated insight into Aboriginal young peoples’ experiences of working alongside Elders and in partnership with mental health services. By using these first-person accounts in their entirety, the authors acknowledge the need for services – as well as researchers – to work with the “wholeness” of peoples’ experiences [41,42]. Dividing and compartmentalising such experiences misses the point in terms of the ways in which services shape their programs to support Aboriginal young people seeking help for mental health concerns [22]. The co-researcher experiences included navigating new and challenging power dynamics, receiving support from the research team and fellow young people, bringing Elders and young people together as a cultural way of working, building confidence and capacity, and realising the bigger picture of systemic change and closing the gap. 

### 4.1. Navigating New and Challenging Power Dynamics 

The coming together of Elders, young people, service staff and policy staff into a shared space revealed complex power dynamics [10,13]. Service and policy staff were considered experts due to their knowledge and experience of the mental health sector, and many had seniority within their organisation in executive, leadership or management roles. There was also a considerable age difference between the youth co-researchers and most other stakeholders. Service and policy staff were predominantly non-Aboriginal, and, therefore, afforded significant power and privilege in colonial Australia. Most staff members also held a dominant, Western worldview and worked in a mainstream service context that affirmed their culture, knowledge and worldview. Although the young people were challenged by this group dynamic, they also recognised the importance of working directly with those in positions of power and can make change from top-down, as Shae (S.A.) reveals: “We cannot underestimate the value and richness that Indigenous perspectives, experiences, and ways of working bring to the table”. Service provider’s recognition of the power imbalance at play and their commitment to slowing down, stepping back, making room, and truly listening to the young people’s their voices was essential and demonstrated a new kind of leadership [22]. Hunter (H.C.) supports this when he says that “Collaborating with young people is essential in developing service plans that are orientated towards their needs, while allowing young people to expand their voice and perspective on issues that affect them”.

### 4.2. Elders and Young People Together: A Cultural Way of Working

The youth co-researchers identified Elders and young people working together as a cultural way of working [3,4]. Sharing and providing direction alongside their Elders was a new experience for the co-researchers, and due to the cultural authority of the Elders, it was difficult at times to navigate the space and demonstrate leadership as a young Aboriginal person. In consideration of this, validation from the Elders was very important, and their support gave the young people the confidence to express their opinions and ideas. It has been documented elsewhere that caring for Indigenous youth is central to the role of Elders in Aboriginal communities [27]. Elders putting their faith in, investing time in, and supporting the young people during the project builds their capacity for their ongoing role as leaders within their communities. Ashton’s (A.R.-W.) reflections align with this: “One of the many experiences gained through working with Building Bridges was being able to practice cultural ways of working, through having Elders teach younger generations the needs that the Aboriginal community demands”.

### 4.3. Support from the Research Team and Fellow Young People

The research team’s advocacy for the young people and their voices, and the peer support from their fellow co-researchers, played a key role in helping the young people recognise the value and importance of their contribution. Throughout the project, members of the research team met frequently with the youth co-researcher group to debrief, seek feedback about their experiences, and resolve any concerns. These meetings allowed the research team to stay connected to the needs of the young people and support them in their work with the services. It also allowed the youth co-researchers to develop an identity as a collective group, to share experiences, inspire each other, and benefit from peer learning, all of which—in addition to the cultural authority of Elders—were seen as protective factors when working together with mental health service providers [3,19]. These reflective spaces allowed the research team and youth co-researchers to acknowledge the power dynamics at play, reflect on how it impacted young people’s confidence and participation, and work together to identify solutions and educate staff on how to work with young people as equal partners in co-design.

### 4.4. Confidence and Capacity Building 

Over time, through their engagement in the project, youth co-researchers developed an even deeper sense of why their voices are important and the value of their contributions within the project and more broadly. Throughout the project, young people were held by Nyoongar Elders, who provided cultural leadership along with an unwavering confidence in the young people’s voices [27,41]. They described the experience of sharing their voice as empowering, rewarding, fulfilling and overdue, as reflected in Hunter’s comments: “Through this experience, I have developed insights into mental health, the mental health sector and cooperative ways of working which have been highly valuable. It has allowed me to be educated on mental health issues which affect the Aboriginal community while also refining my ability to give back and assist”. The young people recognised themselves as the next generation of leaders and appreciated the knowledge and experience gained, including new insights into mental health, the sector, research and co-design. Young people expressed their sense of community responsibility and the tools and confidence they now had to help them advance their communities.

### 4.5. Systemic Change and Closing the Gap

The young people’s passion for their voices to be heard is deeply personal. It is contextualised within a history of decisions being made for them as Aboriginal people, rather than with or by them, and strengthening their voices must therefore be understood through a decolonising lens [2,43]. The impact of the high rates of mental health issues and suicides, and limited support, is experienced firsthand within their own lives, families and communities. This is not just a project for them. For the young people, their work with the services goes beyond individual or organisational change; the focus is on changing the system to dismantle structural racism [44,45]. The young people expressed their deep commitment to working in partnership with mental health and other sectors, and the accountability is on service and policy leaders to step up, as highlighted by Shae (S.A.) and Hunter (H.C.) in the following comments. 

(S.A.): “The people accessing these services are our friends, our family and ourselves, hence why projects like Building Bridges are so crucial”.

(H.C.): “I call on services to work with Aboriginal people to integrate a way of working which interacts and values an Aboriginal contribution and perspective”.

Meaningful systemic change is required to close the gap in health and wellbeing outcomes between Aboriginal and non-Aboriginal Australians, and building trusting relationships with the community, understanding Aboriginal people and culture, and working collaboratively with Elders and young people is a new way forward [18]. There is another gap that requires closing and that is the gap between the understanding and expectations of service providers to ensure their services a relevant and culturally safe for Aboriginal young people.

There are ongoing implications shaped by this project. Firstly, the challenge remains for both researchers and service providers to adhere to the wholeness of youth voices to ensure they are heard in an authentic and meaningful way. Self-determination must be enacted, not simply described [46]. In these first-person accounts, young people are asserting their right to self-determination both in academic writing and in their efforts to co-design mental health service change. Consequently, service providers reconsider the role of young people in contributing to the design and development of services to better meet their needs and have a greater respect and appreciation for local knowledge and direct experiences of young people. With this in mind, we ask: “how does the inclusion of Aboriginal youth voices expose dominant structures and discriminatory practices to enable positive changes to occur?” In this regard, further research into cultural factors impacting therapeutic alliance would also be of benefit [47].

### 4.6. Study Limitations and Research Challenges

The engagement of Aboriginal young people in the Building Bridges project is contingent upon their availability. Exclusion criteria also included the provision that young people were not current clients of the partner service organisations [3]. This paper captures the voices of three of these participants though many others have been recruited. Due to the nature of their engagement, not all Aboriginal youth had equal availability due to community, family and employment or study commitments at the time. This is a challenge service providers also face if they wish to engage Aboriginal youth as co-designers to improve their youth mental health provision to Aboriginal people. For the research team, supporting the youth co-researchers was a privilege and a shared learning experience as the dynamics of the service organisations and the co-design efforts were navigated together, reflective of Aboriginal participatory action research approaches [1,2].

## 5. Conclusions

The Building Bridges project demonstrates that when provided with appropriate opportunities and support structures, Aboriginal young people have the capacity and desire to provide valuable and unique knowledge, insight and expertise towards improving mental health services. The results of these efforts are discussed elsewhere, and ongoing research continues to shape the translation and implementation of the project findings [1,21]. Aboriginal young people’s lived experience is an invaluable resource to developing culturally safe, accessible and responsive mental health care and improving mental health outcomes. In our advocacy for the inclusion of Aboriginal young people in mental health service reform, however, it is essential that we refrain from bringing young people into rooms with other stakeholders with the assumption that they now “have a voice” and that their “voice is heard”. Working meaningfully and in partnership with young people requires an understanding of the importance of Aboriginal youth voice through a decolonising lens. Services must invest time and resources in their work with the community, and be committed to building, holding and sustaining trusting relationships. It requires deep listening, a shared commitment for change, and an openness to new ways of working. True leadership on the part of the mental health sector is supporting Aboriginal young people to lead. Initiatives to strengthen Aboriginal youth voice must be highly localised and designed in partnership with local Elders and young people. It is likely that the learnings from the Building Bridges project have implications for other Aboriginal communities, however, this needs to be considered within the context of local communities and their cultural protocols.

## Data Availability

Not applicable.

## References

[1] Wright M., Culbong T., Webb M., Sibosado A., Jones T., Guima Chinen T., O’Connell M. (2023). Debakarn Koorliny Wangkiny: Steady walking and talking using first nations-led participatory action research methodologies to build relationships. Health Sociol. Rev..

[2] Dudgeon P., Bray A., Darlaston-Jones D., Walker R. (2020). Aboriginal Participatory Action Research: An Indigenous Research Methodology Strengthening Decolonisation and Social and Emotional Wellbeing.

[3] Culbong T., Crisp N., Biedermann B., Lin A., Pearson G., Eades A., Wright M. (2022). Building a Nyoongar work practice model for Aboriginal youth mental health: Prioritising trust, culture and spirit, and new ways of working. Health Sociol. Rev..

[4] Wright M., Culbong T., Crisp N., Biedermann B., Lin A. (2019). “If you don’t speak from the heart, the young mob aren’t going to listen at all”: An invitation for youth mental health services to engage in new ways of working. Early Interv. Psychiatry.

[5] Commonwealth of Australia National Standards for Mental Health Services: Standard 3. Consumer and Carer Participation. Australian Commission on Safety and Quality in Health Care. https://www.health.gov.au/resources/publications/national-standards-for-mental-health-services-2010-and-implementation-guidelines.

[6] Head B. (2011). Why not ask them? Mapping and promoting youth participation. Child Youth Serv. Rev..

[7] Crooks C., Snowshoe A., Chiodo D., Brunette-Debassige C. (2013). Navigating between rigour and community-based research partnerships: Building the evaluation of the uniting our nation”s health promotion program for FNMI youth. Can. J. Commun. Ment. Health..

[8] Kirmayer L., Sheiner E., Geoffroy D., Gau M. (2016). Chapter 6—Mental Health Promotion for Indigenous Youth.

[9] Duley P., Botfield J., Ritter T., Wicks J., Brassil A. (2017). The Strong Family Program: An innovative model to engage Aboriginal and Torres Strait Islander youth and Elders with reproductive and sexual health community education. Health Promot. J. Aust..

[10] Vujcich D., Thomas J., Crawford K., Ward J. (2018). Indigenous youth peer-led health promotion in Canada, New Zealand, Australia, and the United States: A systematic review of the approaches, study designs, and effectiveness. Front. Public Health..

[11] Larsson I., Staland-Nyman C., Svedberg P., Nygren J., Carlsson I. (2018). Children and young people’s participation in developing interventions in health and well-being: A scoping review. BMC Health Serv. Res..

[12] Littleton C., Reader C. (2022). To what extent do Australian child and youth health, and education wellbeing policies, address the social determinants of health and health equity?: A policy analysis study. BMC Public Health.

[13] Canas E., Lachance L., Phipps D., Birchwood C. (2019). What makes for effective, sustainable youth engagement in knowledge mobilization? A perspective for health services. Health Expect.

[14] Australian Bureau of Statistics Causes of Death, Australia, 2017: Intentional Self-Harm in Aboriginal and Torres Strait Islander People. Cat. No. 3303.0. https://www.abs.gov.au/ausstats/abs@.nsf/Lookup/by%20Subject/3303.0~2017~Main%20Features~Intentional%20self-harm%20in%20Aboriginal%20and%20Torres%20Strait%20Islander%20people~10.

[15] Azzopardi P., Sawyer S., Carlin J., Degenhardt L., Brown N., Brown A., Patton G. (2018). Health and wellbeing of Indigenous adolescents in Australia: A systematic synthesis of population data. Lancet.

[16] Westerman T. (2010). Engaging Australian Aboriginal youth in mental health services. Aust. Psychol..

[17] Wright M., O’Connell M., Jones T., Walley R., Roarty L. (2015). Looking Forward Aboriginal Mental Health Project: Final Report.

[18] Commonwealth of Australia (2020). Closing the Gap Report 2020.

[19] Allen J., Mohatt G., Beehler S., Rowe H. (2014). People awakening: Collaborative research to develop cultural strategies for prevention in community intervention. Am. J. Community Psychol..

[20] Corney T., Cooper T., Shier H., Williamson H. (2022). Youth participation: Adultism, human rights and professional youth work. Child Soc..

[21] Wright M., Brown A., Dudgeon P., McPhee R., Coffin J., Pearson G., Lin A., Newnham E., King Baguley K., Webb M. (2021). Our journey, our story: A study protocol for the evaluation of a co-design framework to improve services for Aboriginal youth mental health and well-being. BMJ Open.

[22] McCalman J., Fagan R., McDonald T., Jose S., Neal P., Blignault I., Askew D., Cadet-James Y. (2022). The availability, appropriateness, and integration of services to promote indigenous Australian youth wellbeing and mental health: Indigenous youth and service provider perspectives. Int. J. Environ. Res. Public Health..

[23] South West Aboriginal Land and Sea Council South West Aboriginal Land and Sea Council. http://www.noongar.org.au/.

[24] Iseke J. (2013). Spirituality as decolonizing: Elders Albert Desjarlais, George McDermott, and Tom McCallum share understandings of life in healing practices. Decolonization Indig. Educ. Soc..

[25] Wright M., O’Connell M. (2015). Negotiating the right path: Working together to effect change in healthcare service provision to Aboriginal peoples. Action Learn. Action Res. J..

[26] Wright M., Culbong M., Jones T., O’Connell M., Ford D. (2013). Making a difference: Engaging both hearts and minds in research practice. Action Learn. Action Res. J..

[27] Busija L., Cinelli R., Toombs M., Easton C., Hampton R., Holdworth K., Macleod A., Nicholson G., Nasir B., Sanders K. (2020). The role of elders in the wellbeing of a contemporary Australian indigenous community. Gerontologist.

[28] Absolon K., Willett C., Brown L., Strega S. (2005). Putting Ourselves Forward: Location in Aboriginal Research.

[29] Kovach M., Carriere J., Barrett M., Montgomery H., Gillies C. (2013). Stories of Diverse Identity Locations in Indigenous Research. Int. J. Qual. Res..

[30] Rigney L.-I. (2017). Indigenist Research and Aboriginal Australia. Indigenous Peoples’ Wisdom and Power.

[31] Wain T., Sim M., Bessarab D., Mak D., Hayward C., Rudd C. (2016). Engaging Australian Aboriginal narratives to challenge attitudes and create empathy in health care: A methodological perspective. BMC Med. Educ..

[32] Kovach M. (2021). Indigenous Methodologies: Characteristics, Conversations, and Contexts.

[33] Snijder M., Wagemakers A., Calabria B., Byrne B., O’Neill J., Bamblett R., Munro A., Shakeshaft A. (2020). “We walked side by side through the whole thing”: A mixed-methods study of key elements of community-based participatory research partnerships between rural Aboriginal communities and researchers. Aust. J. Rural Health.

[34] Morton Ninomiya M., Pollock N. (2017). Reconciling community-based Indigenous research and academic practices: Knowing principles is not always enough. Soc. Sci. Med..

[35] MacDonald C. (2012). Understanding participatory action research: A qualitative research methodology option. Can. J. Action Res..

[36] Minkler M., Wallerstein N. (2011). Community-Based Participatory Research for Health: From Process to Outcomes.

[37] Wallerstein N. (1999). Power between evaluator and community: Research relationships within New Mexico’s healthier communities. Soc. Sci. Med..

[38] Wright M. (2011). Research as intervention: Engaging silenced voices. Action Learn. Action Res. J..

[39] Wright M., Lin A., O’Connell M. (2016). Humility, inquisitiveness, and openness: Key attributes for meaningful engagement with Nyoongar people. Adv. Ment. Health.

[40] Teixeira S., Augsberger A., Richards-Schuster K., Martinez L. (2021). Participatory research approaches with youth: Ethics, engagement, and meaningful action. Am. J. Community Psychol..

[41] Gone J., Blumstein K., Dominic D., Fox N., Jacobs J., Lynn R., Martinez M., Tuomi A. (2017). Teaching Tradition: Diverse Perspectives on the Pilot Urban American Indian Traditional Spirituality Program. Am. J. Community Psychol..

[42] Haffey S., Rowland L. (2015). The importance of first person accounts in education: Teacher and student perspectives. Schizophr. Bull..

[43] Dudgeon P., Bray A., Walker R., Page A., Stritzke W. (2020). Chapter 12—Self-determination and strengths-based Aboriginal and Torres Strait Islander suicide prevention: An emerging evidence-based approach. Alternatives to Suicide: Beyond Risk and Toward a Life Worth Living.

[44] Sherwood J. (2013). Colonisation—It’s bad for your health: The context of Aboriginal health. Contemp. Nurse..

[45] Sherwood J., Edwards T. (2006). Decolonisation: A critical step for improving Aboriginal health. Contemp. Nurse..

[46] Dudgeon P., Scrine C., Cox A., Walker R. (2017). Facilitating empowerment and self-determination through participatory action research: Findings from the national empowerment project. Int. J. Qual. Meth..

[47] van Benthem P., Spijkerman R., Blanken P., Kleinjan M., Vermeiren R., Hendriks V. (2020). A dual perspective on first-session therapeutic alliance: Strong predictor of youth mental health and addiction treatment outcome. Eur. Child Adolesc. Psychiatry.

